# Knowledge, Perception, and Willingness to Use Telepharmacy Among the General Population in Indonesia

**DOI:** 10.3389/fpubh.2022.825554

**Published:** 2022-05-11

**Authors:** Nesqi N. Tjiptoatmadja, Sofa D. Alfian

**Affiliations:** ^1^Pharmacist Professional Education Study Program, Faculty of Pharmacy, Universitas Padjadjaran, Jatinangor, Indonesia; ^2^Department of Pharmacology and Clinical Pharmacy, Faculty of Pharmacy, Universitas Padjadjaran, Jatinangor, Indonesia; ^3^Center of Excellence in Higher Education for Pharmaceutical Care Innovation, Universitas Padjadjaran, Jatinangor, Indonesia

**Keywords:** telepharmacy, knowledge, willingness, perception, Indonesia

## Abstract

**Introduction:**

COVID-19 emerged as a pandemic in early 2020. Various steps were taken in an attempt to decrease the spread, which resulted in limited mobility. As people were dissuaded from going out, multiple numbers of digitalized pharmacy services arose to fulfill people's needs for medicine. The objective of this study was to assess knowledge, perception, and willingness to use telepharmacy services and the affecting factors among the general population in Indonesia.

**Patients and Methods:**

A cross-sectional study was conducted with the inclusion criteria of Indonesian citizenship, living in Indonesia, and agreement to participate. Details of demographic characteristics, knowledge and perception of telepharmacy services and willingness to use them were collected using an online questionnaire that was adapted from a previous study. The results were analyzed using a descriptive analysis method. The associations between demographic characteristics and knowledge, perception, and willingness to use telepharmacy services were tested with the Mann–Whitney U Test.

**Results:**

Of 203 participants participated in this study, 51% of them had heard about telepharmacy. Over 98% of the participants had a positive perception of telepharmacy services. The majority of those who had never used it were willing to try telepharmacy services in the future. Age and educational level were significantly associated with knowledge of telepharmacy services. No associations were observed between demographic characteristics and perception and willingness to use telepharmacy services.

**Conclusions:**

General population in Indonesia had a fair knowledge, a positive perception, and were willing to use telepharmacy services. Interventions to increase knowledge of telepharmacy in Indonesia need to target older adults and people who are less educated.

## Introduction

In late December 2019, cases of illness with pneumonia-like symptoms arose in Wuhan, China. By January 2020, the World Health Organization (WHO) concluded that the disease was caused by a novel coronavirus which was later named Sars-COV-2. The WHO announced that the outbreak was a public health emergency of international concern by January 30th, 2020, making it a pandemic (1).

COVID-19 spread quickly in over 210 countries (2). In March 2020, the first case of COVID-19 was detected in Indonesia (3). As the number of cases rapidly increased, the government enforced various measures to control the spread of the virus. The measures included the obligation to wear a mask, self-quarantine when one felt unwell, physical distancing, and later on, the requirement to get vaccinated (4, 5). The Indonesian government has been issuing restrictions on communities' activities since July 2021 and these have been prolonged until the beginning of 2022 (6). The measures resulted in limited mobility and made it difficult for face-to-face pharmacy counseling to take place. Furthermore, medical facilities in Indonesia were overloaded as the cases soared (7).

To overcome this, the Indonesian government issued a policy to implement telemedicine by July 6th, 2021 (4). Telemedicine is defined as health care services that are performed from a distance using technology and include an exchange of information on diagnosis, treatment, and prevention (8). Telepharmacy, as one type of telemedicine, provides remote pharmaceutical services including drug counseling, self-medication, drug monitoring, and evaluation by a qualified pharmacist (9).

Telepharmacy is a tool that can be used to reach underrepresented populations and thereby ensure equitable pharmaceutical services (10). Telepharmacy services in the United Arab Emirates can improve patient access to health care providers, ease the health care burden, and reduce dispensing errors (11). In the Republic of Srpska, Bosnia and Herzegovina, telepharmacy was used most for consulting on chronic disease, followed by consulting about COVID-19, and about acute diseases (12).

A previous study conducted in Jordan including 364 community pharmacists as participants showed that 91% of them agreed that telepharmacy helps patients to get faster medical feedback (13). Another studies reported that most pharmacy students at the University of Tennessee Health Science Center and one University in Jordan were not familiar with telepharmacy but thought that it would be useful to prevent medication error, save time (14) and they showed a positive willingness to use the services (13). The factors affecting people's choice to use telepharmacy in Indonesia are the regulations, the technology used by the patients, and financial status (15). However, evidence about knowledge and perception of telepharmacy and willingness to use it among people in Indonesia remains unclear. This information is important to develop interventions to improve the acceptability of telepharmacy.

The objective of this study is to assess awareness, perception, and willingness to use telepharmacy services and the affecting factors among the general population in Indonesia.

## Materials and Methods

### Study Design and Setting

An observation cross-sectional survey was conducted among the general population in Indonesia. The data were collected from October 7th, 2021 to October 15th, 2021 from participants who met the inclusion criteria: being an Indonesian citizen, currently living in Indonesia, and agreeing to participate. We did not restrict the inclusion criteria based on participants' demographic characteristics such as age or length of residency to capture the general population in Indonesia. The Health Research Ethics Committee of Universitas Padjadjaran, Indonesia approved the study protocol (No. 967/UN6.KEP/EC/2021).

### Procedure

The participants were randomly approached online through social media (Twitter, Instagram, and Facebook) and groups chat in smartphone applications. Data about knowledge and perception of telepharmacy services and willingness to use them were collected using an online questionnaire (Google Forms) in Indonesian language that was based on a previously published study (16). The questionnaire consisted of four sections: demographic characteristics, knowledge, perception, and willingness to use telepharmacy.

#### Demographics

This section explores the diversity of the participants using questions on age, gender, level of education, and province of origin.

#### Knowledge About Telepharmacy Services

Knowledge of telepharmacy was assessed using several questions including, “Do you have a chronic disease?,” “Have you ever heard about telepharmacy?,” and “Have you ever used a telepharmacy service?”

#### Perception of Telepharmacy Services

This section is to assess participant's perception of the telepharmacy services to agree or not with some statements including, “There are many telepharmacy services that I can use in Indonesia.” “I like using the telepharmacy services,” “The telepharmacy service is important to be able to communicate with medical practitioners whenever and wherever,” “The telepharmacy service helps to save energy and time,” “The telepharmacy service helps to cut service costs,” “I am willing to pay for telepharmacy services,” and “I will recommend telepharmacy services to my family and friends.” The questions' ratings for perception were measured using a five-point Likert-type scale ranging from 1 = strongly disagree to 5 = strongly agree. The perception groups were divided into two which were defined a priori as participants with total score of 1–17 being grouped as having poor perception, whereas participants with total score of 18–35 being grouped as having good perception.

#### Willingness to Use Telepharmacy Services

The participants who had not used telepharmacy before were assessed about their willingness to use it in the future by asking the question, “If not, are you interested in using telepharmacy?”

### Sample Size Calculation

Using the Slovin formula (17, 18) to determine the minimum sample size, a minimum of 100 participants was required to obtain a 95% confidence level and a margin of error of 10% based on the Indonesian total population of 273 million (19).

### Data Analysis

Descriptive statistics were used to summarize the participants' characteristics. The normality test was conducted using the Kolmogorov–Smirnov test since the sample size was higher than 50 participants. The associations between demographic characteristics and knowledge, perception, and willingness to use telepharmacy services were tested with the Mann–Whitney U Test since all data were not normally distributed. All statistical analyses were carried out using SPSS software (version 25.0; IBM, Armonk, NY, USA).

## Results

### Demographic Characteristics

A total of 203 participants participated in this study (response rate 99.5%). The majority of the participants were aged around 20–30 years old (*n* = 120, 58.8%) and female (*n* = 139, 68.6%). Most participants came from West Java (*n* = 143, 70.1%), followed by East Java (*n* = 12, 5.9%), Banten (*n* = 10, 4.9%), and Central Java (*n* = 10, 4.9%) (Table 1). Most participants did not report having a chronic disease (*n* = 181, 89.9%).

**Table 1:** Demographic characteristics of the participants (*N* = 203).

	***N***	**Percentage (%)**
**Age (years)**		
<15	3	1.5
16–19	22	10.8
20–30	120	58.8
31–40	28	13.7
41–50	18	8.8
51–60	12	5.9
>60	1	0.5
**Gender**		
Male	64	31.5
Female	139	68.5
**Level of education**		
Middle school	5	2.5
High school	81	39.7
Associate's degree	10	4.9
Undergraduate	91	44.6
Master's degree	10	4.9
Doctoral degree	5	2.5
Others	2	1
**Province of origin**		
Aceh	4	2
Banten	10	4.9
Bengkulu	3	1.5
Central Java	10	4.9
DI Yogyakarta	2	1
DKI Jakarta	8	3.9
East Java	12	5.9
East Kalimantan	1	0.5
Lampung	1	0.5
Maluku	1	0.5
North Sumatera	2	1
Riau	2	1
South Sulawesi	1	0.5
South Sumatera	1	0.5
West Java	143	70.1
West Kalimantan	3	1.5

### Knowledge, Perception, and Willingness to Use Telepharmacy Services

For the section on participants' knowledge about telepharmacy, 181 participants (89.6%) had no chronic illness and 104 participants (51%) had heard of telepharmacy (Table 2). Most of them (*n* = 162, 79.8%) had never used the telepharmacy services, but 89.9% of them (*n* = 160) were interested in using it (Table 2).

**Table 2:** Knowledge and willingness to use telepharmacy services.

**Questions**	**N (%)**	
	**Yes**	**No**
**Knowledge about telepharmacy services**		
Have you ever heard about telepharmacy?	104 (51.0%)	99 (49.0%)
Have you ever used telepharmacy service before?	41 (20.2%)	162 (79.8%)
**Willingness to use telepharmacy services**		
If not, are you interested in using telepharmacy services?	160 (89.9%)	18 (10.1%)

Seventy-six participants (37.4%) felt neutral about the statement “there are many different telepharmacy services I can use in Indonesia” (Table 3). The second question was only for those who had used telepharmacy services and 68 out of 146 participants (46.6%) felt neutral about the statement on liking to use a telepharmacy service. The third question was about the importance of telepharmacy for communicating with medical practitioners whenever and wherever in which 92 participants (45.3%) agreed. The participants strongly agreed that the telepharmacy service is important in saving time and energy. Of them, 86 participants (42.4%) also strongly agreed that the telepharmacy service helps in cutting service costs and 82 participants (40.4%) were willing to pay for the service. Lastly, 79 participants (38.9%) were willing to recommend telepharmacy services to their family and friends (Table 3).

**Table 3:** Perception of telepharmacy services.

**Statements**	**Answer (%)**				
	**Strongly disagree**	**Disagree**	**Neutral**	**Agree**	**Strongly agree**
There are many telepharmacy services that I can use in Indonesia	2 (1%)	25 (12.3%)	76 (37.4%)	69 (34%)	31 (15.3%)
I like using telepharmacy service	3 (2.1%)	6 (4.1%)	68 (46.6%)	47 (32.2%)	22 (15.1%)
Telepharmacy service is important to be able to communicate with medical practitioner whenever and wherever	0 (0%)	1 (0.5%)	25 (12.3%)	92 (45.3%)	85 (41.9%)
Telepharmacy helps to save time and energy	0 (0%)	1 (0.5%)	23 (11.3%)	83 (40.9%)	96 (47.3%)
Telepharmacy helps to reduce service costs	2 (1%)	6 (3%)	49 (24.1%)	60 (29.6%)	86 (42.4%)
I am willing to pay for telepharmacy services	7 (3.4%)	16 (7.9%)	65 (32%)	82 (40.4%)	33 (16.3%)
I will recommend telepharmacy to my friends and family	1 (0.5%)	7 (3.4%)	53 (26.1%)	79 (38.9%)	63 (31%)

### Factors Associated With Knowledge Perception, and Willingness to Use Telepharmacy Services

Age (*p*-value = 0.000) and educational level (*p*-value = 0.031) were significantly associated with knowledge about telepharmacy services (Table 4). No association was observed between gender and knowledge of telepharmacy services.

**Table 4:** Association between demographic characteristics and knowledge, perception, and willingness to use telepharmacy.

	**(*p*-value)**
**Knowledge**	
Age	0.000
Gender	0.912
Level of education	0.031
**Perception**	
Age group 1	0.245
Age group 2	0.634
Gender group 1^*^	0.669
Gender group 2^#^	0.663
Level of education group 1^*^	0.435
Level of education group 2^#^	0.982
**Willingness**	
Age	0.589
Gender	0.664
Level of education	0.536

* *Group 1: Participants who had used telepharmacy services*.

# *Group 2: Participants who had never used the telepharmacy services*.

In the perception section, the results were divided into participants who had used telepharmacy services (Group 1) and participants who had never used the telepharmacy services (Group 2). There were no associations between age, gender, educational level and perception about telepharmacy services among these two groups (Table 4).

There were no associations observed between age (*p*-value = 0.589), gender (*p*-value = 0.664), educational level (*p*-value = 0.536) and willingness to use the telepharmacy services (Table 4).

## Discussion

Over half of the 203 participants in our study had heard about telepharmacy services. Most participants had a good perception of the telepharmacy services that were offered. Participants who had never used a telepharmacy service also showed an interest in using it in the future. Age and educational level were significantly associated with knowledge of telepharmacy services. No associations were observed between demographic characteristics and perception and willingness to use telepharmacy services.

This study showed that 51% of the participants had heard about telepharmacy. This number is higher than a study conducted in India (18.9%) (16). Furthermore, 89.9% of the participants who had never used telepharmacy services in our study were willing to use them in the future. This result is in line with a previous finding that telemedicine usage s has been increasing since the pandemic occurred (20). It was supported by the fact that although 89.6% of our participants are healthy, they have heard of telepharmacy before. The reason might be the restrictions that have been implemented in Indonesia; which resulted in people using more online services, such as telepharmacy, to fulfill their daily needs.

Despite the limited knowledge, we observed that 98% of the participants had a positive perception of telepharmacy services. They agreed that telepharmacy would benefit them in terms of cutting time, energy, and cost. They also believed that the service would be beneficial to give them more flexibility because it is performed online. This finding is supported by a previous study that the patients who received telemedicine health care were very satisfied with the service they had received (21). The usage of telemedicine has been proven to enhance the quality of care for patients with diabetes in Saudi Arabia by successfully monitoring and maintaining their blood glucose levels (22).

In this study, we observed that age is significantly associated with knowledge about telepharmacy services in Indonesia. This can be explained by the different amounts of exposure to technology in each group. The younger groups tend to be more familiar with the newer technology, which results in them being more aware of the newer services that have been offered. In another study that was conducted in the United States, older people were not likely to be interested in using telemedicine services due to their lack of belief that the health care purpose can be achieved at a distance (23).

Participants who have higher education in our study seem to be more aware of telepharmacy services. Similarly, this was due to people with higher education being exposed to more technology. They are also more likely to pay more attention to the newest regulations that have been issued by the Indonesian government. This finding is also supported by another study in Egypt that showed highly educated people have more knowledge of telemedicine services (24). We further observed no association between gender and knowledge about telepharmacy services. This may be due to digitalization where everyone has access to the internet regardless of their gender.

No association was observed between gender, age, and educational level with the perception of telepharmacy services in our study might be due to the awareness of participants about the importance of maintaining their health. There was adequate exposure from health promotion programs through social media and counseling to everyone regardless of their age, gender, or educational level. Although no association was observed between age, gender, or level of education and willingness to use telepharmacy services, we observed that most participants are willing to use the services.

Our findings implied that there is still a gap between the knowledge about telepharmacy the participants have within different age and educational level groups. A more thorough approach should have been given to older people and people who are less educated by offline counseling to ensure that people who are not so familiar with technology can get the same exposure. Furthermore, a health promotion program should target the patients' families for supporting older people. The health promotion program can also be conducted in education and health facilities to ensure that everyone knows the newest regulations regarding telepharmacy services.

To our knowledge, this is the first thorough evaluation of knowledge, perception, and willingness to use telepharmacy, as well as factors associated with it, among Indonesian participants. However, some limitations need to be mentioned. Data collection using online questionnaire has several methodological drawbacks: certain populations are less likely to have internet access and to respond to online questionnaire, the lack of a trained interviewer to clarify the information provided can lead to less reliable data, and voluntary participation can result in participants with biases selecting themselves into the sample. We also could not draw causal inferences regarding the temporal associations between age and educational level with knowledge of telepharmacy services. Furthermore, most of the participants in our study were university students and without chronic diseases, thus, possibly not representative of the general population. Therefore, we advise caution in interpreting and extrapolating our results. Future studies should focus on qualitative research to provide a more in-depth understanding of the acceptance of telepharmacy services. Furthermore, a better understanding of the knowledge and perception of telepharmacy services and willingness to use it of different age, location (urban vs. rural) and comorbidity groups over a longer period including all provinces would give a more comprehensive overview of telepharmacy practices in Indonesia. Such findings might support research on how to develop effective interventions and acceptance of telepharmacy practices.

## Conclusions

Of the 203 participants in our study, most of them had a fair knowledge and had positive perceptions about the telepharmacy service. Those who had never used it were also interested in using telepharmacy in the future. Interventions to increase knowledge of telepharmacy in Indonesia need to target older adults and people who are less educated.

## Data Availability Statement

The raw data supporting the conclusions of this article will be made available by the authors, without undue reservation.

## Ethics Statement

The studies involving human participants were reviewed and approved by the Health Research Ethics Committee of Universitas Padjadjaran. The patients/participants provided their written informed consent to participate in this study.

## Author Contributions

NT conceived and designed the study, performed the study, analyzed and interpreted the data, and wrote the paper. SA conceived and designed the study and analyzed and interpreted the data. Both authors contributed to the article and approved the submitted version.

## Conflict of Interest

The authors declare that the research was conducted in the absence of any commercial or financial relationships that could be construed as a potential conflict of interest.

## Publisher's Note

All claims expressed in this article are solely those of the authors and do not necessarily represent those of their affiliated organizations, or those of the publisher, the editors and the reviewers. Any product that may be evaluated in this article, or claim that may be made by its manufacturer, is not guaranteed or endorsed by the publisher.
